# Research progress on biodegradable polymer-based drug delivery systems for the treatment of knee osteoarthritis

**DOI:** 10.3389/fbioe.2025.1561708

**Published:** 2025-04-10

**Authors:** Jinchi Zhang, Jinchao Zhang, Hailong Li, Huimin Zhang, Hongyan Meng

**Affiliations:** ^1^ Department of Medical, Qingdao Binhai University, Qingdao, China; ^2^ Department of Nursing, The Third People’s Hospital of Heze, Heze, China

**Keywords:** biodegradable polymers, knee osteoarthritis, drug delivery system, treatment, synthetic polymers, natural polymers

## Abstract

Knee osteoarthritis (KOA) is a disease that involves multiple anatomical and physiological changes in the knee tissues, including cartilage degeneration, bone remodelling and formation of bony encumbrances, which leads to clinical manifestations including pain, stiffness, swelling and limitation of knee function. Knee osteoarthritis is a chronic joint disease characterised by degenerative cartilage lesions and secondary osteophytes in the knee joint. The symptoms of knee osteoarthritis tend to progress slowly, and at this stage, the number of patients with KOA is increasing. However, due to the adverse effects and poor therapeutic outcomes following surgical treatment, intervention therapy through the utilisation of biodegradable polymeric materials is required. Currently, clinical aspects are mainly used to treat cartilage degeneration in patients with osteoarthritis of the knee by using different kinds of biodegradable biopolymer materials with excellent physical properties, histocompatibility and other properties, combined with a drug delivery system, which can reduce the level of inflammation and stiffness in the focal area, and maximise the restoration of the patient’s knee joint joint mobility and athletic ability. Based on the properties of the polymeric material drug delivery system, the polymeric material has a variable drug loading capacity that encapsulates hydrophobic/hydrophilic drugs and controls the release kinetics by regulating the composition and charge. This paper reviews the research progress of Poly (ε-caprolactone) (PCL), Poly(lactic acid) (PLA), Poly (lactic glycolic acid) (PLGA), Poly(ethylene glycol) (PEG) synthetic polymers and collagen, chondroitin sulfate, other natural polymers based drug delivery systems for the treatment of knee osteoarthritis, and explains that different biodegradable polymeric materials have been widely used for the treatment of knee osteoarthritis. However, there are still issues of degradability, toxicity, compatibility, and durability and safety of the drug delivery system of degradable materials that need to be addressed in further clinical trials. As biodegradable biomedical materials continue to be explored, eventually idealized polymeric materials will stand out in the treatment of KOA.

## Graphical Abstract

**Figure:**
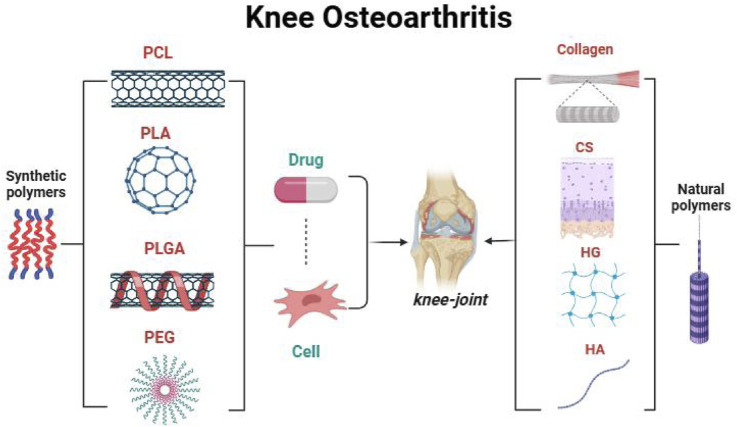
Application of different polymers in the treatment of knee osteoarthritis.

## 1 Introduction

Knee osteoarthritis (KOA), also known as deformable knee arthritis, it is a common degenerative chronic disease of the knee joint (Amin et al., 2020). The main pathological features of KOA include degenerative changes in the articular cartilage, formation of a bone capsule through reactive hyperplasia of the articular margins and cartilage, and eventual destruction of the articular surfaces of the knee joint. Its prevalence increases with age, with no gender difference, and is statistically higher in women than in men aged 65 years or older (Mine et al., 2019). Clinical results show that KOA is very common in the elderly, and for people over 50 years old, the incidence of KOA is second only to coronary heart disease and stroke, which seriously affects the patient’s ability to perform daily life and quality of life, and increases the burden on the family. Statistics show that KOA may become the fourth most common disease leading to disability in China (Zhu et al., 2018; Buechele et al., 2018). The prevalence of symptomatic knee osteoarthritis (SxOA) was 40% higher in Beijing patients than in women in Framingham, USA (Zhang et al., 2001). A population-based cohort study conducted in the United Kingdom reported that the all-cause mortality rate of patients with osteoarthritis of the knee was about 55 percent higher than that of the general population (Nüesch et al., 2011).

At this stage, the clinical use of surgery to treat patients with knee osteoarthritis patients, the therapeutic effect is better. Han et al. (2021) treated 90 patients with knee osteoarthritis patients through periprosthetic osteotomy of the knee, and the study showed that periprosthetic osteotomy of the knee has a high degree of safety, and it can significantly improve the degree of joint pain, joint mobility, and quality of life of patients with KOA. However, surgical treatment will exist *in vivo* cartilage tissue lesions, muscle strength and elasticity around the tendon decreases, resulting in difficulty in bearing external impact and pressure around the knee joint. In recent years, biodegradable polymer materials have been widely used in the treatment of knee osteoarthritis, based on the excellent properties of biodegradable polymer materials to improve the ideal material carrier for the regeneration of repaired degenerative cartilage. Chitosan (CS) is a natural polymer material with good biodegradability, antimicrobial and osteogenic properties, and is now widely used in the repair and treatment of bone tissues. Wang et al. (2024) investigated the advances of CS-based composites, such as micro- and nano-delivery carriers, hydrogels, coating materials, and polymeric materials of bone tissue-engineered scaffolds for the treatment of KOA, which provided the development to supply some theoretical support. The current status of natural and synthetic polymer nanoparticles (PNPs) for drug delivery and KOA therapy has also been discussed, and studies have shown that the biodegradability and surface modification of the nanocarriers positively correlate with drug release profiles and tissue targeting (Rahimi et al., 2021). Duro-Castano et al. (2018) have developed a fluorescence resonance energy transfer peptide probe that can be cleaved by matrix metalloproteinase-13 (MMP-13), which was subsequently further coupled to a 30 kDa linear oligoglutamate chain. The results of the study indicate that the polymeric material, using the drug as a carrier, can effectively block the degradation of cartilage and degenerative lesions during the development of KOA, and that this new polymeric probe can be used as an effective clinical tool for the detection of early osteoarthritis of the knee. Current clinical treatments include allografts, autografts, medications, glucocorticoids, and surgery. However, these treatments work primarily to contribute to cartilage repair, but not in cartilage regeneration. Tian et al. (2020) observed the effect of treatment by establishing a rabbit knee osteoarthritis model and using polyester fibre bandage in combination with a plastic rod at 75 degrees of knee flexion. Pathological results showed that the model group had typical pathological changes of knee osteoarthritis, and the group Mankin score was significantly higher than the control group. The study shows that the use of polymer bandage combined with plastic rod can successfully establish the rabbit knee osteoarthritis model, with strong stability of the local joints and controllable movement angle of the knee joint. Aulin et al. (2023) investigated the carbazate-modified polyvinyl alcohol (PVAC) *in vitro* and human chondrocyte cell cultures by Western blot in a rat osteoarthritis model, and investigated whether PVAC alone could be used to treat knee osteoarthritis in the rat. The anti-inflammatory effect of PVAC was investigated in rat osteoarthritis model by constructing a rat model of osteoarthritis of the knee and examining the efficacy of PVAC alone in the treatment of knee osteoarthritis. The study showed that *in vivo* PVAC localised to cell membranes and prevented acrolein-induced cell death, and that mechanically abnormal pain, as measured by von Frey filaments, was reduced at medium to high PVAC concentrations. This study demonstrates that PVAC has been shown to have a protective effect on soft tissue and reduce pain responses. Bhosale et al. (2024) used thermal homogenisation to amplify lornoxicam loaded nanostructured lipid carriers (LRX-NLCs) loaded with clonoxicam after optimisation, LRX relieves pain and inflammation associated with knee osteoarthritis. F14 LRX-NLCs were combined with carbopol 940 LR gel to form LRX-NLCs-Gel and their efficacy was studied in SD rats. The study demonstrated a significant decrease in the levels of pro-inflammatory cytokines such as interleukin 1β (IL-1β) and tumour necrosis factor-α (TNF-α) *in vivo*, and a significant decrease in cartilage regeneration and inflammatory response with LRX-NLCs-Gel. Similarly, Kang et al. (2020) and others rationally designed acid-activated curcumin polymers (ACP) as therapeutic pre-drugs of curcumin, in which curcumin was covalently doped into the backbone of amphiphilic polymers and used in a mouse model of osteoarthritis of the knee. It was shown that ACP polymer micelles greatly protect knee joint structures from arthritis by inhibiting TNF-α and IL-1β. Seo et al. (2022) used injectable polymer nanoparticle-based hydrogels loaded with triamcinolone acetonide (TCA) for the treatment of KOA.They synthesised amphiphilic polyorganophosphonitrile with temperature-dependent nanoparticle formation and sol-gel transition behaviour when dissolved in aqueous solution and delivered TCA in a rat model of osteoarthritis of the knee. It was found that TCA-encapsulated polymer nanoparticles strongly inhibited the inflammation of knee osteoarthritis in rats without any adverse effects. This study demonstrated that TCA can be used as a long-acting adrenocorticotropic hormone analogue with effects on glucose metabolism, anti-inflammatory, anti-toxic, anti-shock and anti-allergic effects. Katyal et al. (2022) also made it a candidate for injectable polymers for the treatment of osteoarthritis of the knee in rabbits through the use of E_5_C recombinant protein block polymers, which were able to form a porous reticular gel at physiological temperatures. It was shown that the E_5_C gel prolonged the release of Atsttrin protein derivatives and inhibited chondrocyte catabolism, whilst facilitating anabolic signalling *in vitro*, and reduced the expression of local inflammatory factors in the knee joint.

It has been further investigated by further research into the pathology of knee osteoarthritis, but delivering therapeutic agents to the target site with low invasiveness, high retention and low side effects remains a challenge (Lin et al., 2022). However, both current and new clinical therapies can benefit from targeted approaches that selectively deliver drugs to the knee at therapeutic concentrations while limiting systemic drug exposure (Mei et al., 2021).

Therefore, there is a need for a “compensatory substance” that can treat osteoarthritic diseases. Tissue engineering techniques and clinical rehabilitation can be used as an alternative to current treatments to help cartilage repair and cartilage regeneration (Jain and Ravikumar, 2020).

This review of the most widely studied biodegradable polymer materials, including PCL, PLA, PLGA, PEG, and other polymer materials, summarises the progress of biodegradable polymers in the treatment of osteoarthritis of the knee. It can provide technical support for the replacement of cartilage tissue in the clinic, and also develop biodegradable polymer materials with excellent properties to promote the regeneration of cartilage tissue around the knee joint, so that patients can return to their families and the society as soon as possible, and reduce the economic burden.

## 2 Synthetic polymer-based drug delivery systems

### 2.1 Effect of PCL on the treatment of knee osteoarthritis

PCL is a semi-crystalline polymer, which is a chemically synthesised biodegradable polymer materials with five non-polar methylene groups on the structural repeating unit - CH_2_ starch and other substances are co-mingled to produce fully biodegradable materials. PCL is now widely used in the treatment of chronic diseases such as osteoarthritis of the knee due to its good degradability properties and histocompatibility.

**FIGURE 1:**
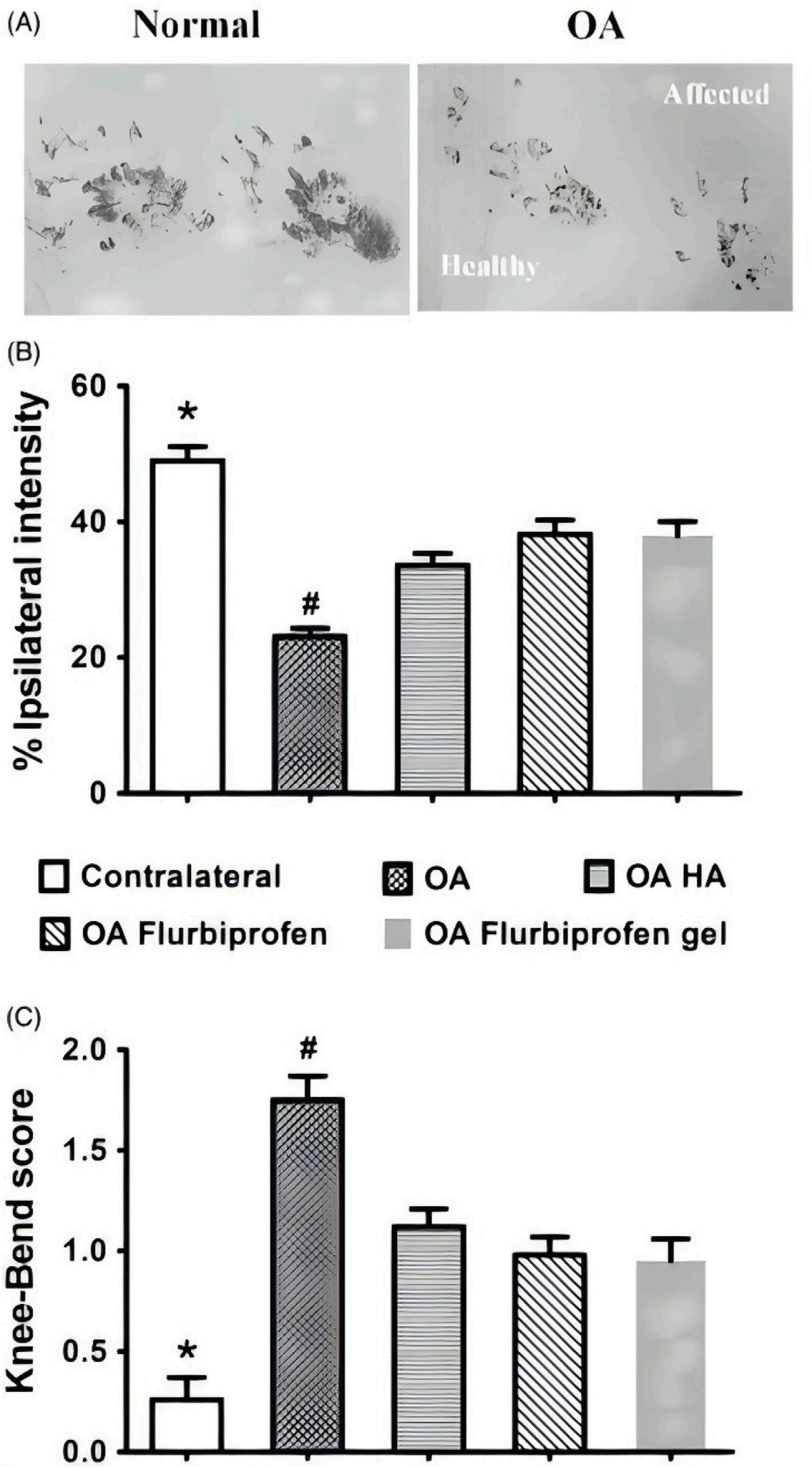
**(A)** Flurbiprofen thermal gel has similar short-term analgesic effect on OA rats. Based on the CatWalk test of paw print intensity on the contralateral healthy side and the ipsilateral affected OA side (affected), the **(B)** TIPPI percentage and **(C)** knee flexion scores of the four experimental groups before and 2 h after the first dose of the drug. CC BY 4.0. Copyright 2020 The Author(s).

Vahedi et al. (2019) performed induction of chondrogenesis and promotion of cartilage repair *in vivo* through the development of a polycaprolactone nanofibre scaffold with stem cells as a carrier for application to the infrapatellar fat pads of sheep’s knees. The results of the study showed that morphological assessment revealed that the defects in the adipose tissue-derived stem cells (ASCs)/PCL scaffold group were completely filled with cartilage-like tissue, while the other groups showed the formation of a thin layer of cartilage-like tissue in the defect. Toluidine blue staining of the repaired tissues in the ASCs/PCL group showed proliferation of chondrocytes, with markedly increased clusters of chondrocytes, lateral chondrocytes were still dividing, and lateral chondrocyte tissue was not yet complete, indicating that the polycaprolactone polymer promoted chondrogenesis of infrapatellar adipose tissue-derived stem cells *in vivo*, providing theoretical support for the treatment of knee osteoarthritis. Liang et al. (2020) prepared nanofibrous films consisting of PCL and PCL-grafted-lignin (PCL-g-lignin) copolymers by solvent-free ring-opening polymerisation reaction for the treatment of rabbit KOA model. Lignin provides intrinsic antioxidant activity while PCL tailors its mechanical properties. The results showed that PCL-g-lignin nanofibrous membrane inhibits reactive oxygen species (ROS) generation and activates antioxidant enzymes as well as attenuates inflammation levels through autophagy mechanism, which was observed to be biocompatible and biodegradable through arthroscopy and capable of providing sustained antioxidant activity. Additionally, It has also been shown that by developing a novel, three-dimensionally (3D) printed PCL scaffold and filling the scaffold with mesenchymal stem cells (MSCs), it will be applied to the treatment of osteoarthritis of the knee in New Zealand Large White rabbits. It was found that the scaffold containing mesenchymal stem cells group had a significantly better gross appearance, white glistening, and smooth surface compared to the cell-free group. Fibrochondrocytes with collagen types I, II and III and proteoglycans were found in both the MSC-containing scaffold group and the cell-free scaffold implant at 12 and 24 weeks, while the MSC-containing scaffold group showed significantly better results at 24 weeks. In addition, the MSC-containing scaffold group had significantly better mechanical properties in both compared to the cell-free group. Implantation of bone marrow MSCs in PCL scaffolds increases the regenerative capacity and mechanical strength of their fibrocartilage tissue and provides a favourable means of efficiently treating knee osteoarthritis (Zhang et al., 2017). Other scholars have used the same technique to develop a cartilage regeneration system based on CS hydrogel/3D-printed PCL hybrids with recruitment of tetrahedral framework nucleic acid (TFNA) injected into the joint cavity for the treatment of knee osteoarthritis in rabbits. The results showed that the 3D-printed PCL scaffold provided basic mechanical property support, and the composite scaffold was a promising material for promoting cartilage regeneration based on chitosan-targeted TFNA recruitment and TFNA-enhanced synovial mesenchymal stem cells (SMSCs) proliferation and chondrogenesis (Li et al., 2021). As the same as above, Cao et al. (2021) demonstrated the feasibility of composite scaffolds to promote cartilage regeneration by preparing composite scaffolds consisting of methacrylated alginate (ALMA) co-mingled with PCL and testing their morphological analysis and mechanical properties. Bone marrow mesenchymal stem cells (BMSCs) loaded gradient PCL/ALMA scaffolds exhibited good cell survival and proliferation, and type II collagen fibronectin deposition. Such composite scaffolds applied for the treatment of knee osteoarthritis have good clinical application prospects and do not produce adverse reactions to the organism. At this stage, drug therapy has become a commonly used treatment in the clinic, which can effectively reduce the inflammatory factors around the knee joint due to the slow sustained release property of the drug. Li et al. (2020) designed a PCL-b-poly (ethylene glycol)-b-PCL (PCL-PEG-PCL) triblock copolymer flurbiprofen thermosensitised gel for the treatment of osteoarthritis of the knee in SD osteoarthritis of the knee in rats. *In vitro* drug release studies demonstrated sustained release of flurbiprofen from the thermal gel for more than 3 weeks. The CatWalk test was used to evaluate the effect of improving OA-induced dyskinesia in rats based on the strength of the footprints (Figure 1A); The results of the study, expressed as a percentage of the total strength of the ipsilateral footprints in the sum of the two footprints, were 33.6 ± 1.76 in the hyaluronate (HA) group, 38.2 ± 2.07 in the flurbiprofen group, and 37.9 ± 2.07 in the flurbiprofen gel after 2 h of treatment group was 37.9 ± 2.14 (Figure 1B); The results of the knee flexion test were consistent with a decrease in response scores from 1.75 ± 0.12 to 1.12 ± 0.09 in the CatWalk group in the HA group, and to 0.98 ± 0.09 in the flurbiprofen group and 0.95 ± 0.11 in the flurbiprofen gel group (Figure 1C). This study suggests that the thermosensitive copolymer PCL-PEG-PCL is suitable for the sustained intra-articular effects of flurbiprofen in knee osteoarthritis and has great potential in the treatment of knee osteoarthritis.

Tawfeek and Esaily (2022) prepared collagen-coated PCL nanofibres and examined them by SEM. Mononuclear cells from peripheral blood and synovial fluid were cultured with human bone marrow MSCs stimulated with phytohemagglutinin using two co-cultures (MSCs/nano and MSCs/nano) and used for the treatment of knee osteoarthritis. The study demonstrated the novel function of nanofibres to enhance the immunomodulatory effects of MSCs on osteoarthritis by increasing the expression of intercellular adhesion molecules (ICAM) for the treatment of symptoms of knee osteoarthritis. Liu et al. (2021) used a bone marrow-derived mesenchymal stem cell (BMSC)-loaded 3D bioprinted multilayered scaffold containing methacrylated hyaluronic acid (MeHA)/PCL for the treatment of articular cartilage defects in the knee bone of rats. The study demonstrated that the BMSC-loaded scaffold promoted cartilage formation by promoting collagen II and inhibiting interleukin 1β in the femoral talocalcaneal cartilage defects, and that the loaded scaffolds also had good biomechanical properties. This study demonstrates the potential of 3D bioprinted BMSC-loaded scaffolds in suppressing joint inflammation and promoting repair of articular cartilage defects in KOA.

Unlike the above, Abdelhamid et al. (2023) used a novel surgical technique of 3D bioprinted micronised adipose tissue (MAT) grafts for the treatment of arthritic cartilage defects in human knee joints and assessed the extent of admixture of such graft types by arthroscopic and radiological analysis. Ten patients received 3D bioprinted grafts composed of MAT and allogeneic hyaline cartilage matrix on polycaprolactone moulds and were monitored until 12 months postoperatively. The results of the study showed that at 12 months postoperatively, using the cartilage repair tissue magnetic resonance observational scores improved to a mean of 82.85 ± 11.49, and we observed complete union of the grafts with the surrounding cartilage. This would be groundbreaking research, the first application of biodegradable polymer materials in humans with good biocompatibility, and would be a novel regenerative technique for the clinical management of patients with osteoarthritis of the knee. In addition, Gu et al. (2022) prepared three integrated scaffolds by selective laser sintering with different non-channel, continuous channel and discontinuous channel modes, and the cartilage and subchondral regions of the integrated scaffolds consisted of small PCL microspheres and large PCL microspheres, respectively, which were used for repairing cartilage in rabbit osteoarthritis of the knee. The study demonstrated that the discontinuous channel scaffold had an integrated hierarchical structure, adaptable compressive strength, and gradient interconnecting porosity, which provided an effective renewable microsphere scaffold for cartilage reconstruction in knee osteoarthritis.

In conclusion, the influence of PCL on the treatment of knee osteoarthritis is mainly reflected in its excellent performance as a biomedical material. By promoting the repair and regeneration of cartilage and meniscus and reducing the occurrence of postoperative complications, PCL provides new ideas and solutions for the treatment of knee osteoarthritis. Through the PCL scaffold, chondrocytes can be guided to proliferate and differentiate at the defect site to form new cartilage tissue, thus filling the defect and restoring the normal structure and function of the joint.

### 2.2 Effect of PLA on the treatment of knee osteoarthritis

PLA is a biodegradable polymer which has a wide range of applications in the medical field, including in the treatment of osteoarthritis of the knee. However, when directly discussing the impact of PLA on the treatment of knee osteoarthritis, it is necessary to clarify the specific manner in which it is applied, as PLA is usually not used directly as a single drug or treatment, but as a component of scaffolding materials or other composites that play a role in, for example, tissue-engineered cartilage reconstruction in knee osteoarthritis.

**FIGURE 2:**
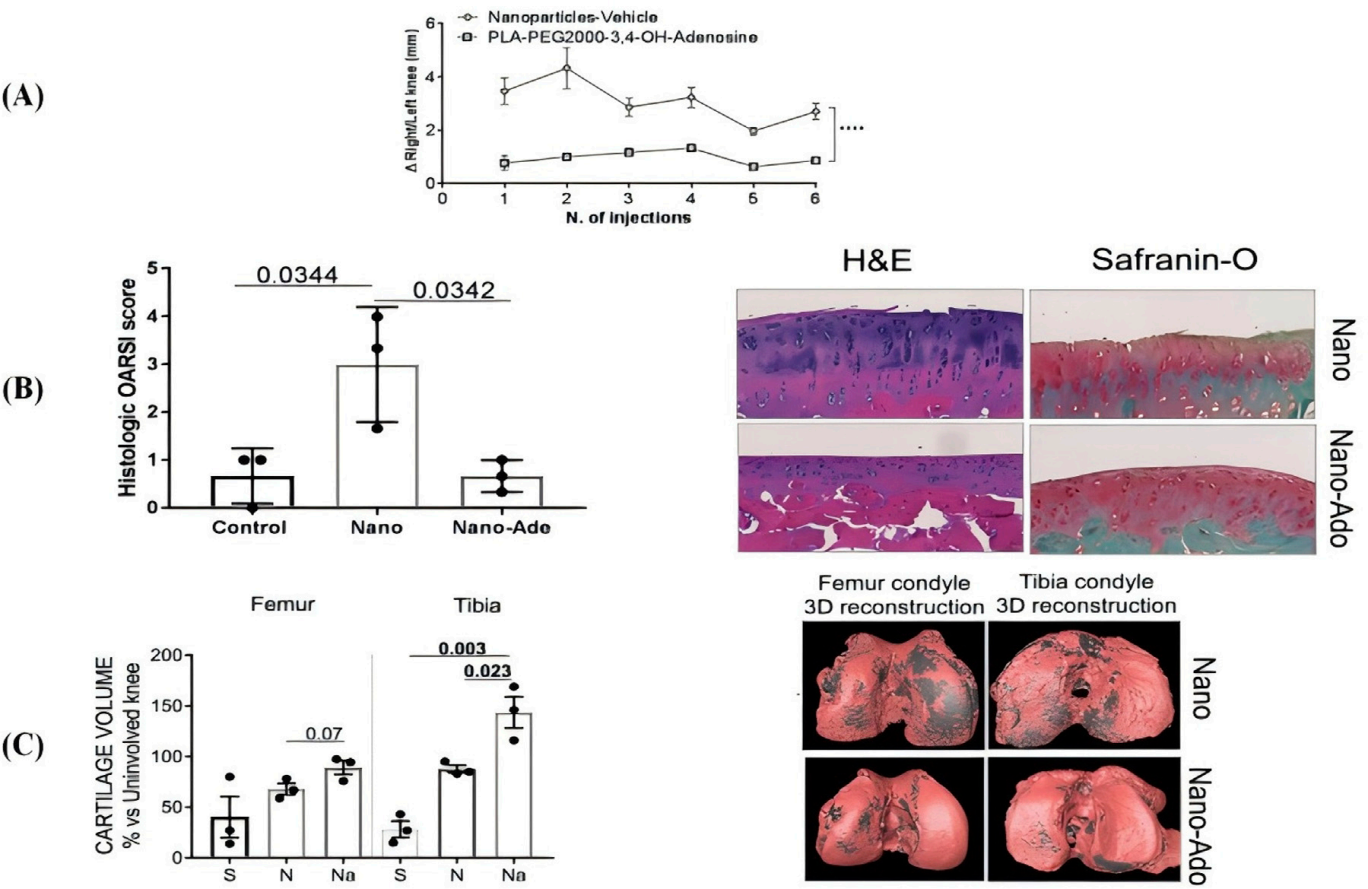
**(A)** Intra-articular injection of adenosine-conjugated NPs reduces knee joint swelling; **(B)** Histological analysis shows less cartilage protection, proteoglycan loss and reduced OARSI scores in nanoAdo-treated rats compared to rats treated with the carrier; **(C)** Reconstruction of the µCT data reveals a reduction in cartilage surface damage. CC BY 4.0. Copyright 2019 The Author(s).

Liu X. et al. (2019) reported the synthesis and activity of six targeted polymeric PEG-b-PLA nanoparticles as couplers of adenosine receptor ligands by detecting their ability to stimulate the increase of cAMP in RAW264.7 mouse macrophages using click chemistry. Adenosine nanoparticles were tested *in vivo* in a rat model of post-traumatic knee osteoarthritis, and intra-articular injection of adenosine-coupled nanoparticles attenuated swelling in the affected knee joints, while uncoupled nanoparticles had no effect on knee swelling (Figure 2A). Compared to adenosine-coupled nanoparticles, rats using adenosine-coupled rats showed a significant reduction in cartilage surface oscillations by hematoxylin and eosin staining (H&E), and Safranin-O staining showed a reduced loss of proteoglycans, leading to a significant reduction in Osteoarthritis Research Society International (OARSI) scores (Figure 2B). Compared with uncoupled nanoparticles, intra-articular injection of adenosine-coupled nanoparticles in the knee joint prevented the loss of cartilage, and these reconstructed pink soft tissues were observed in micro-CT images (Figure 2C). The experimental results showed that intra-articular injection of adenosine nanoparticles in the knee joint prevented the development of osteoarthritis in this model.

**FIGURE 3:**
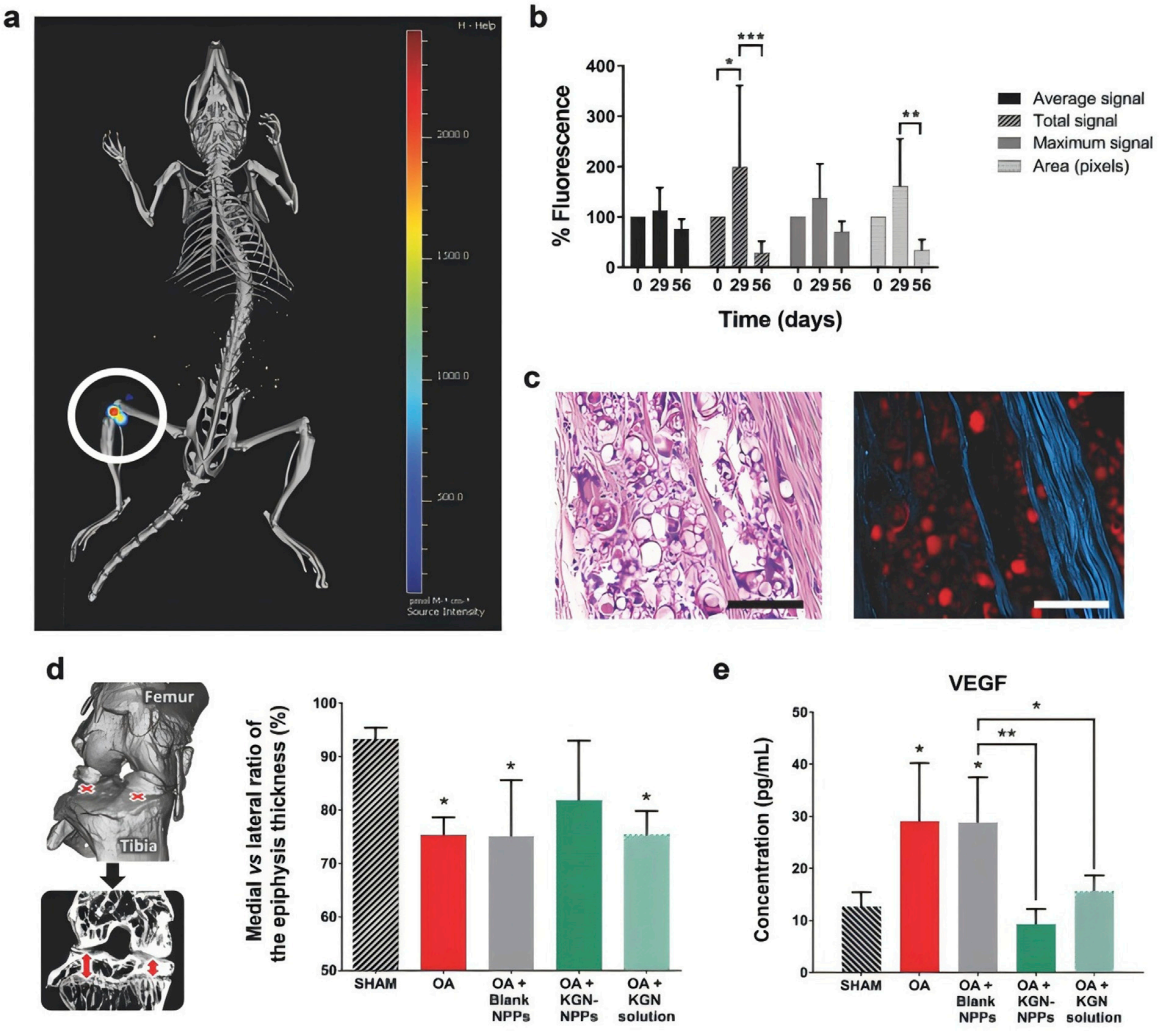
**(a)** Representative *in vivo* fluorescence superimposed on an X-ray image of a mouse (Source intensity ranges from 0 to 2,450 pmol m^−1^ cm^−1^); **(b)** Percentage of *in vivo* fluorescence in mice normalised after IA injection of KGN-NPPs; **(c)** Representative H&E paraffin-embedded tissue light (right) and fluorescence (right) micrographs of mouse lateral knee KGN-protein; **(d)** Percentage ratio of medial to lateral tibial epiphyseal thickness at Day 56; **(e)** Multiplexed enzyme-linked immunosorbent assay (ELISA) analysis of VEGF in mouse plasma at Day 56. CC BY 4.0. Copyright 2018 The Author(s).

Abou-ElNour et al. (2019) synthesised novel derived polymers from PLA and poly-delta-polylactic acid (PDL) to produce microparticles (MPs) derived from the novel sustainable polymers loaded with triamcinolone acetonide (TA) for the treatment of rheumatoid knee osteoarthritis via intra-articular (IA) delivery. Solubility studies demonstrated high compatibility between TA and PEG-PDL1700 and blending with PLA facilitated the formation of MPs. Evaluating the rate of inflammation inhibition and histopathology in the knee joints of rats induced by complete Fuchs’ adjuvant, the study demonstrated the advantages of using sustained polymers for effective delivery of the drug in new combinations with good therapeutic efficacy for the treatment of osteoarthritis of the knee in rats. Maudens et al. (2018a) studied a fluorescent polymer, polylactide, as an internal tracking particle, which was developed by combining Cyanine 7 (Cy7) covalently linked to PLA and in turn developed nanocrystal-polymer particles (NPPs). The nanocrystals were produced from kartogenin (KGN), a wet-milled nucleoprotein known to promote regeneration of cartilage in osteoarthritis of the knee, and a fluorescent biodegradable polylactic acid polymer for *in vivo* particle tracking was synthesised to treat osteoarthritis of the knee in mice. The results of the study showed that *in vivo* fluorescence of KGN-NPPs superimposed on X-rayed bone from a representative mouse confirmed high local retention within the joint over a two-month period (Figure 3a); The duration of the NPPs was monitored by Cy7 fluorescence semiquantitative *in vivo* analyses over a 2-months period (Figure 3b); Photograph of paraffin-embedded mouse lateral knee KGN protein after HE staining (Figure 3c); On the 56th day, representative hematoxylin and eosin paraffin-embedded tissues were seen on the lateral knees of the mice photomicrographs and fluorescence micrographs (Figure 3d); The percentage of medial and lateral tibial epiphyseal thickness was measured using micro-computed tomography imaging, and the effect of KGN-NPP treatment on vascular endothelial growth factor was more pronounced compared with KGN solution, as detected by multiplex ELISA (Figure 3e). The experiments demonstrated the significant therapeutic effect on knee osteoarthritis using the extended drug delivery system for the treatment of knee osteoarthritis in humans.

Salama et al. (2020) prepared bioadhesive hybrid nanoparticles loaded with etoricoxib for the treatment of osteoarthritis with anti-inflammatory and bone reconstructive efficacy by using solvent evaporation technique for the preparation of emulsion with PLA and chitosan hydrochloride and Captex^®^ 200 as liquid oil, polyvinyl alcohol (PVA) and Tween^®^ 80 as surfactants. Liang et al. (2022) grafted PLA into lignin by ring-opening polymerisation followed by electrostatic spinning, varying the lignin content in the system was able to adjust the physicochemical properties of the resulting nanofibres for the treatment of osteoarthritis of the knee in SD rats. *In vitro* studies showed that PLA-lignin nanofibres protected BMSCs from oxidative stress and promoted chondrogenic differentiation. *In vivo* studies demonstrated that lignin nanofibres could promote cartilage regeneration and repair cartilage defects within 6 weeks after implantation. This study demonstrates that lignin-based nanofibres can be used as antioxidant tissue engineering scaffolds to promote cartilage regeneration for KOA therapy. In addition, Nakagawa et al. (2021) performed repair of rabbit knee osteoarthritic menisci by creating horizontal tears and wrapping them with sheet scaffolds containing polyglycolic acid, polylactic acid and polycaprolactone. The results of the study showed that the regenerative effect of the wrapped group was significantly better than that of the horizontal group, especially at 16 weeks (P < 0.05), and the wrapped treatment induced fibrochondrocyte-like cells most significantly at 16 weeks. This study demonstrates that restoration of meniscal function by wrapping polymer treatment prevents cartilage degeneration and symptoms of knee osteoarthritis. Some scholars have fabricated cannabidiol-loaded poly(lactic acid-hydroxyacetic acid copolymer) copolymers (CBD-PLGA) nanoparticles (NPs) for the repair of KOA in rats.The study demonstrated that CBD-PLGA-NPs copolymers significantly inhibited the expression of inflammatory cytokines by serum lipase (LPS)-induced primary rat chondrocytes, and effectively protected against LPS-induced damage to cell viability. damage. The prepared CBD-PLGA-NPs showed good protection of primary chondrocytes *in vitro* and can be used as an effective material for the treatment of KOA (Jin et al., 2023). Niazvand et al. (2017) induced monoiodoacetic acid in rat KOA using curcumin-containing poly(lactic acid)-hydroxyacetic acid copolymer acid nanoparticles and monoiodoacetic acid was injected into the right knee joint to induce osteoarthritis. The results of the study showed a significant increase in cytoarchitecture and matrix staining of articular cartilage in curcumin-treated animals compared to the monoiodoacetic acid group. The study demonstrated that nano-curcumin administration blocked the inflammatory response to KOA in rats. Unlike the above, Poly(L-lactide acid) (PLLA) is a bioabsorbable degradable material with good biocompatibility and biodegradability. PLLA is made by polymerisation of L-lactic acid monomers, which has been widely used in tissue engineering scaffolds, bone fixation and bone repair. It has been widely used in tissue engineering scaffolds, bone fixation and bone repair. Murakami et al. (2017) established a novel cell-free meniscus scaffold containing polyglycolic acid (PGA) or PLLA for knee meniscus repair in the great white rabbit. It was shown that the sponge PLA significantly increased the intensity of iNOS staining and the number of lymphocytes at 8 weeks, and the intensity of PLLA staining at 12 weeks. Fan et al. (2022) used solution blending method to prepare bone-conducting poly(L-lactide-ran-p-dioxanone-ran-glycolide) (PLPG)/nanohydroxyapatite (nHAP) (P/H) composites, and 3D printing technique to prepare P/H scaffolds for repairing soft tissues of knee joints. In addition, the same approach has been used to introduce poly (di-lactic acid) (PDLA) into PLPG to enhance its crystalline properties by *in situ* generation of stereocomplexed poly (lactic acid) (SC-PLA). It was shown that PLPG/PDLA composites have good processability, while the scaffolds have good mechanical and degradation properties. PLPG/PDLA scaffolds have an important potential for application in bone tissue engineering, and the PLPG scaffolds also have controlled degradation rates and mechanical properties, which can effectively promote the regenerative capacity of bone tissues (Fan et al., 2024; Fan et al., 2023). Li J. et al. (2024) synthesised poly(trimethylene carbonate-lactide-glycolide) (PTLG) copolymers by ring-opening polymerisation of PLLA and glycolic acid monomers by being heated and dissolved in ethyl acetate, followed by natural cooling and recrystallisation for removal of impurities and water. It was shown that PTLG vascular scaffolds have a bionic surface morphology with parallel microgrooves and ridge structures, which are highly similar to the inner surface of human blood vessels in terms of structure and parameters, and promote cell adhesion growth. It suggests that PTLG vascular scaffolds have the potential to be applied for KOA repair. The medial collateral ligament (MCL) has also been regenerated using absorbable scaffolded PLLA scaffolds in a rabbit model. The MCL defect was surgically created in the knee and then reconstructed using a scaffolded PLLA scaffold. The study showed that a thin layer of fibrocartilage was observed at the ligament-bone junction at 8 and 16 weeks and that the fibre arrangement became denser at 16 weeks. It was shown that PLLA scaffolds allow regeneration of the MCL with type I collagen expression and fibrocartilage formation and can be used as a potential polymer scaffolding material for KOA repair (Nishimoto et al., 2012).

Therefore, in the treatment of knee osteoarthritis, PLA scaffolds are used to provide space for chondrocyte growth and differentiation and promote the regeneration and repair of cartilage tissue by tissue engineering methods. This scaffold material is able to guide cells to grow in specific locations and form new cartilage tissue, thereby improving the symptoms of patients with knee osteoarthritis. PLA can also be combined with other materials to improve the mechanical properties and biocompatibility of the scaffold, and composites have shown good application prospects in tissue-engineered cartilage reconstruction for knee osteoarthritis.

### 2.3 Effect of PLGA on the treatment of knee osteoarthritis

Nowadays, the impact of PLGA on the treatment of KOA is mainly reflected in its application as a drug carrier or loaded with stem cells. As a polymer materials with good biocompatibility and biodegradability, PLGA has been widely used in the development of drug slow-release systems, including drug delivery in the treatment of osteoarthritis of the knee. Stem cells are loaded into PLGA microspheres or nanoparticles, and the drug is delivered to the lesion site, including by joint cavity injection. In addition, PLGA loaded carriers can control the rate of release and the extent of distribution of the stem cells, allowing for sustained action of the stem cells within the joint.

**FIGURE 4:**
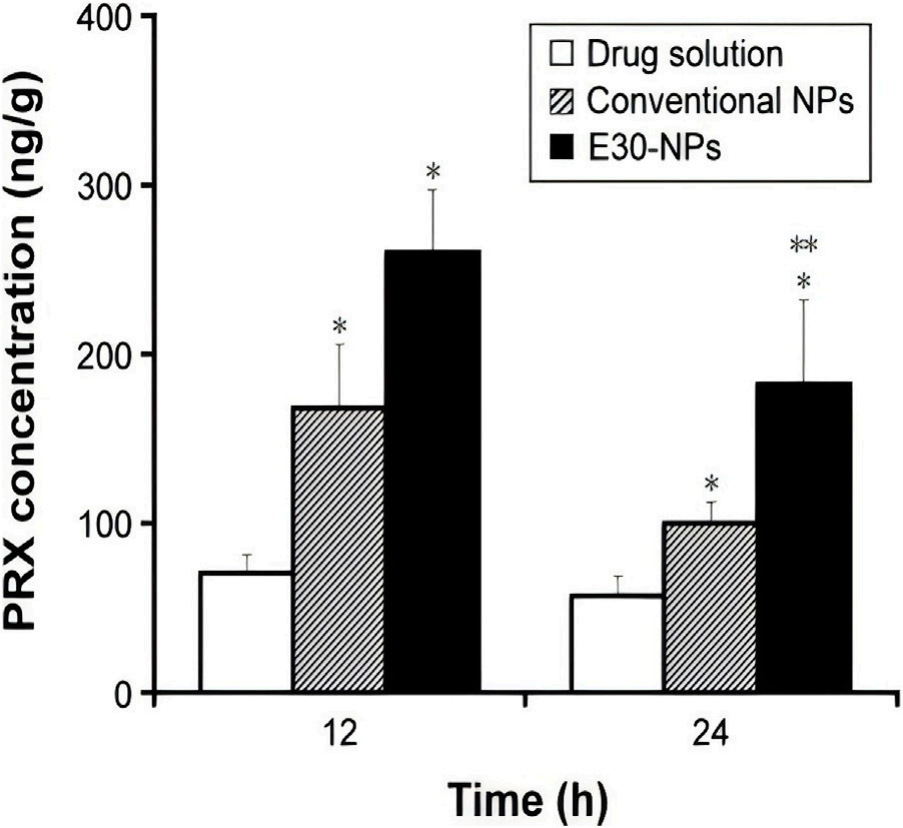
IA drug solutions, conventional NPs and E30-NPs dosed at 0.2 mg/kg administered at 12 and 24 h.Note: Vertical bars indicate mean ± standard deviation (n = 6). Statistical analysis was performed using the StudentStudentt test (*P < 0.05 for drug solutions; **P < 0.05 for conventional NPs). E30-NPs, NPs prepared with PLGA and Eudragit RL. CC BY 4.0. Copyright 2016 The Author(s).

Higaki et al. (2005) injected PLGA-nanosteroids intravenously in rats with osteoarthritis of the knee by preparing betamethasone sodium phosphate (BSP) from 100 to 200 nm PLGA nanoparticles. The results showed that a single injection of 30 mg of PLGA-nanosteroids resulted in almost complete relief of the inflammatory response after 1 week. In contrast, free BSP after three administrations of the same dose only modestly reduced the severity of inflammation. In the same vein as above, the same approach was also used to develop betamethasone sodium phosphate/PLGA nanospheres containing water-soluble corticosteroid sodium phosphate, whereby a suspension of nanospheres loaded with BSP was injected into the joint cavities of a rabbit model of antigen-induced arthritis, which was evaluated by measuring joint swelling. *In vitro* release studies showed sustained release of the drug for more than 3 weeks. In rabbits with antigen-induced arthritis, joint swelling was significantly reduced by administration of BSP-loaded nanospheres during 21 days after intra-articular stimulation. It illustrates the effectiveness of drug-loaded nanospheres in the treatment of knee osteoarthritis and confirms the occurrence of polymer degradation in the synovial membrane stimulated through steroidal action of the drug (Horisawa et al., 2002). Kamel et al. (2016) showed a significant reduction in the swelling of the joints in rabbits with osteoarthritis by selecting modified self-assembled nano-systems (SANS) containing glucosamine for the *in vivo* study of osteoarthritis of the knee in albino rats because of the sustained drug release profile and the small particle size. The results showed that both knee joint diameter and TNF-α levels were significantly reduced in the formulations containing PLGA and glucosamine, suggesting that SANS with PLGA and glucosamine can be effective in the treatment of knee osteoarthritis. In addition, Kim et al. (2016) achieved an increase in the retention time of Piroxicam (PRX) in the joint by piroxicam-loaded nanoparticles consisting of PLGA and polyvinyl alcohol by forming micrometre-sized electrostatic clusters in the synovial lumen with endogenous HA in order to reduce systemic exposure after IA. In animal experiments, knee joint tissues were collected to estimate the level of PRX in the joints. The concentration of PRX in the injected tissues decreased rapidly after IA administration. Only 0.3% and 0.2% were detected at 12 h and 24 h after administration, respectively (Figure 4).

Zerrillo et al. (2019) generated PLGA nanoparticles and PH-responsive PLGA-NPs. NPs loaded with HA as a possible model drug for KOA with the use of near-infrared(NIR) dyes and compared in in vitro, *in vivo* and *ex vivo* experiments. The experimental results showed that both NPs treatments appeared to induce a reduction in KOA progression, with the PH-responsive NPs showing a more pronounced effect. PLGA-NPs containing HA and ammonium bicarbonate are candidates for the treatment of osteoarthritis of the knee. Mota et al. (2019) constructed an alternative delivery system based on HA hydrogels and oleic acid PLGA particles for the treatment of osteoarthritis of the knee in mice. It was shown that the HA conjugation efficiencies of oleic acid-free and oleic acid-containing PLGA particles were respectively 73.6% and 86.2%, and the *in vitro* HA release from PLGA particles showed sustained characteristics. A higher therapeutic effect was observed for the drug delivery system of PLGA particles loaded with HA compared to HA solution. Meanwhile, it has also been shown that by combining PLGA and Pluronic F127 (PF127), more stable and uniformly sized nanoparticles can be obtained than PLGA or PF127 alone (Wang et al., 2015).

**FIGURE 5:**
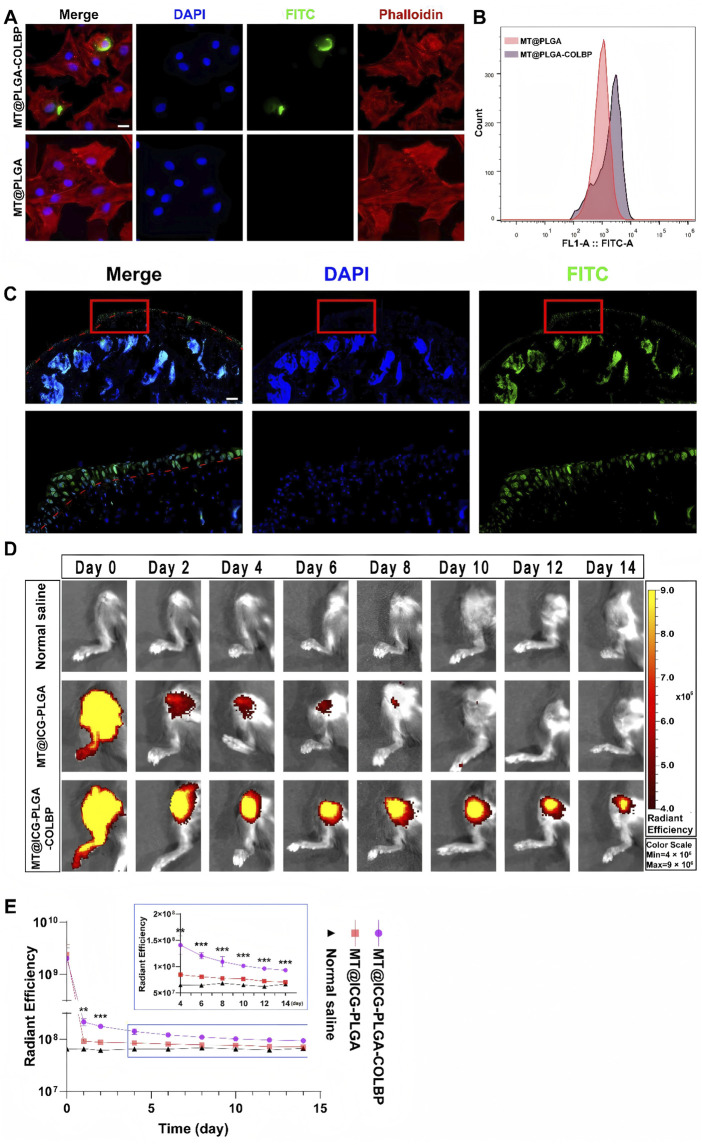
**(A)** Representative confocal images of MT@PLGA and MT@PLGA-COLBP in chondrocytes; **(B)** Detection of FITC-labelled COLBP signals in chondrocytes by flow cytometry; **(C)** Penetration of MT@PLGA-COLBP in free mouse knee joints; **(D)** IVIS photographs of MT@ICG-PLGA and MT@ICG-COLBP in mouse knees; **(E)** Analysis of ROI fluorescence intensity in IVIS images. Data are expressed as SD ± mean: **p < 0.01, ***p < 0.001. CC BY 4.0. Copyright 2023 The Author(s).

In contrast to the above, a novel polymer microsphere (MS) containing trace amounts of TA has been constructed to prolong intra-articular drug retention after IA in the knee joint, and thus to treat knee osteoarthritis. This new system prolonged drug retention in the rat joint, providing quantifiable TA retention for more than 28 days, and studies have shown that TA microcrystals loaded with MS are expected to be beneficial for particular patients with osteoarthritis of the knee and to reduce the frequency of IA administration (Ho et al., 2019). Rezaee-Tazangi et al. (2020) demonstrated the efficacy of curcumin-loaded PLGA nanoparticles (CUR-loaded PLGA NPs) for the treatment of monosodium iodoacetate-induced osteoarthritis of the knee. It was shown that treatment of knee osteoarthritis in the CUR-loaded PLGA NPs group suppressed serum interleukin-1β, tumour necrosis factor-α, interleukin-6, and CUR-loaded PLGA-NPs treatment attenuated the loss of type II collagen as compared to the KOA group. Deng et al. (2024) compared PLGA nanoparticles encapsulated in chondrocyte membranes (CM-NPs), CM-NPs whose internalisation was mainly mediated by E-calmodulin, lattice protein-mediated endocytosis and microcellular drinking for the treatment of knee osteoarthritis in rats. The results of the study revealed that CM-NPs adhered to the cartilage ECM of rat knee joints *in vivo* and penetrated deeply into the cartilage matrix with a residence time of more than 34 days. This study demonstrated the feasibility of using chondrocyte membrane-encapsulated nanoparticles to enhance the pharmacokinetics and efficacy of anti-KOA drugs. Liang et al. (2023) conducted a study by preparing a melatonin-loaded nano-delivery system (MT^@^PLGA-COLPB) and evaluating its behaviour in mouse cartilage and therapeutic effects in KOA. The fluorescence signals of MT@PLGA-COLBP in chondrocytes were observed by fluorescence microscopy and detected by flow cytometry, indicating that the nano-delivery system could enter chondrocytes within 12 h (Figures 5A, B); MT^@^PLGA-COLBP nanoparticles could enter the surface of the articular cartilage within 48 h (Figure 5C); Non-peptide nanoparticles were dissipated on the 10th day post-injection, while the peptide nanoparticle particles were still detectable on day 14 (Figure 5D); The fluorescence signals of MT^@^ICG-PLGA and MT^@^ICG-PLGA-COLBP showed significant differences from the first day after injection (Figure 5E). It was shown that MT^@^PLGA-COLPB could inhibit the activation of the innate immune system by inhibiting the TLR2/4-MyD88-NFκB signalling pathway and scavenging ROS, thereby improving cartilage matrix metabolism and delaying the progression of KOA *in vivo*, suggesting that nanoparticles constituted of PLGA polymer-loaded drugs are promising for the prevention of knee osteoarthritis.

Furthermore, Hamdalla et al. (2022) used solvent evaporation method to encapsulate SM into PLGA constituting nanoparticles (SM-PLGA) against monoiodoacetic acid (MIA)-induced osteoarthritis in rats. The results of the study showed significant improvement in histological criteria, oxidative stress and knee flexion scores in the SM-PLGA group as compared to free SM treated rats. This suggests that PLGA polymer effectively enhances the anti-KOA effect of SM on the knee joint. Morille et al. (2016) induced differentiation of MSCs into chondrocytes for the treatment of pathologic osteoarthritis through the use of microspheres of a novel PLGA-P188-PLGA, and tested microspheres for *in vitro* and *in situ* release of TGF-β3 (PAM-T). The results showed that early injection of MSC inoculated with PAM-T after KOA induction produced protection of cartilage from degradation, and this effect was associated with higher survival of MSCs in the presence of PAM-T. In conclusion, PLGA plays an important role in the treatment of knee osteoarthritis. As a drug carrier, PLGA can achieve sustained release and local high concentration distribution of drugs, improve the therapeutic effect and reduce toxic side effects. With the continuous development of material science and pharmaceutical preparation technology, the application prospect of PLGA in the treatment of knee osteoarthritis will be broader. However, it should be noted that although PLGA sustained release systems have many advantages, their specific application still needs to be individualized according to the specific circumstances of patients and drug characteristics.

### 2.4 Effect of PEG on the treatment of knee osteoarthritis

The direct impact of PEG in therapeutic KOA is not directly in terms of being a therapeutic agent or a drug itself, but rather it may be used as part of a drug carrier or a modified material that indirectly affects therapeutic efficacy by improving the efficiency of drug delivery and release. Modification of PEG can alter the surface properties of the drug and reduce the immunogenicity and clearance of the drug in the body, thus prolonging the circulation time of the drug in the body and improving the bioavailability of the drug.

**FIGURE 6:**
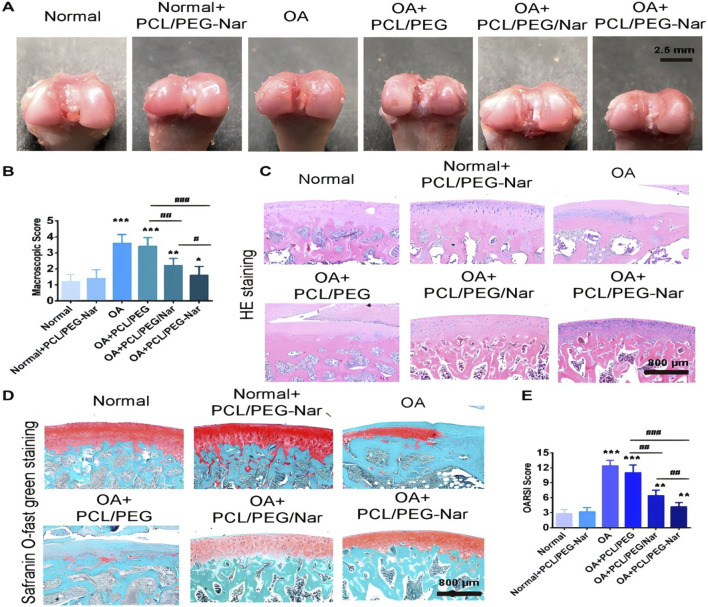
**(A)** Macroscopic observation of cartilage after 4 weeks of PCL/PEG-Nar nanofibre membrane treatment; **(B)** Macroscopic scoring of cartilage after 4 weeks of PCL/PEG-Nar nanofibre membrane treatment; **(C)** H&E staining and **(D)** Saffronin O/fast green staining, Scale bar: 800 μm; **(E)** Evaluation of OARSI score after 4 weeks of PCL/PEG-Nar nanofibre membrane treatment. CC BY 4.0. Copyright 2022 The Author(s).

At the present stage, microparticles or hydrogels can now be designed to improve the administration and intra-articular delivery of specific drugs targeting the molecular pathways and causative mechanisms involved in the progression and remission of osteoarthritis of the knee (Gambaro et al., 2021). IA drug delivery is a common strategy whose therapeutic efficacy is largely dependent on the efficacy of the delivery system used for the treatment of KOA. Different types of IA drug delivery systems, such as microspheres, nanoparticles, and hydrogels, have been rapidly developed over the past decade to improve their therapeutic efficacy (Ma et al., 2023). Current pharmacological treatments for osteoarthritis of the knee focus on relieving the symptoms of pain and inflammation through the use of non-steroidal anti-inflammatory drugs. Liu P. et al. (2019) partially delivered etoricoxib into the articular cavity of the rat knee bone through the use of PLGA-PEG-PLGA triblock copolymer NPs as a drug delivery system. The study showed that etoricoxib-loaded NPs displayed sustained drug release for more than 28 days *in vitro*. Intra-articular injection of etoricoxib-loaded NPs attenuated the inflammatory response in subchondral bone, synovium and cartilage in a rat KOA model. Mok et al. (2020) designed and synthesised methoxy PEG-L-poly (alanine) (mPEG-PA) polymers. Quercetin (Que) is a bioflavonoid with anti-inflammatory and antioxidant properties and is now being used as an oral supplement for KOA. The results of the *in vitro* study showed that chondrocytes were viable after 72 h of mPEG-PA incubation, and the release of Que could continue for more than 28 days. In addition, continuous administration of Que (50 μg) alleviated the progression of osteoarthritis in the knee. Baharizade et al. (2024) prepared self-nanoemulsifying physically crosslinked PEG organogel (SNE-POG) as an innovative hybrid system for topical delivery of insoluble and unstable bioactive compounds and for the treatment of osteoarthritis of the knee in guinea pigs. A double-blind clinical trial confirmed significant reductions in pain scores, stiffness and animal physical function difficulties at the end of 8 weeks in the SNE-POGs group compared to the placebo group, indicating that systemic treatment of osteoarthritis of the knee is very efficient. SNE-POGs show great potential as a novel topical delivery system for water-insoluble and unstable drugs, and could provide a safe effective alternative to conventional topical drug delivery systems. Liu et al. (2024) synthesized a PEG-stabilized bilayer modified cationic liposome(PEG-GS-CLis) as a drug delivery system to achieve the treatment of KOA in rats by delivering glucosamine sulfate (GS) drugs. The results showed that the encapsulation efficiency of PEG-GS-CLis was (96.18 ± 5.77)% and the drug loading capacity was (9.61 ± 0.28)%, which provided a good drug sustained-release effect. The drug delivery system could more effectively reduce the knee bone and joint surface injury and inhibit the expression of inflammatory factors, with good biosafety. In addition, some scholars have designed the PH response system of PCL/PEG-naringenin (PCL/PEG-Nar) nanofibrous membrane by electrospinning technique and used it as a delivery system for the treatment of KOA in rats (Figure 6A); PCL/PEG/Nar nanofibrous membrane treatment had the lowest OA joint macroscopic score (1.60 ± 0.31), which decreased by 55.56% compared with OA group (Figure 6B); HampE staining showed that fibrillation and osteophyte hyperplasia were significantly reduced in OA + PCL/PEG-Nar group after 4 weeks (Figure 6C); Saffron O-fast green showed significant cartilage degeneration, significantly reduced histological lesion severity and increased proteoglycan in OA + PCL/PEG-Nar group (Figure 6D); OARSI showed that OA + PCL/PEG/Nar and OA + PCL/PEG-Nar treatment decreased OARSI score by 48.38% and 77.42%, respectively, compared with OA group (Figure 6E). Studies have shown that OA + PCL/PEG-Nar nanofibrous membranes can cleverly “Open” and continuously release Nar with anti-inflammatory effects in a weak acid KOA microenvironment to reduce the degree of KOA injury (Wang et al., 2022).

Mancipe Castro et al. (2020) by designing nanocomposite 4-arm PEG-maleimide (PEG-4MAL) microgels containing PLGA nanoparticles (NPs) as IA small molecule drug delivery system. Microgels containing rhodamine B drug loaded PLGA-NPs were synthesised by microfluidic technology for the treatment of knee osteoarthritis in male Lewis rats. The results of the study showed that the PEG-4MAL microgel was retained in the knee joint space of rats for at least 3 weeks without inducing any joint degenerative changes. In addition, the PEG-4MAL microgel formulations were all retained in the knee synovium, and they significantly increased the IA retention time of the model small molecule near-infrared dyes compared to the free dyes. It is concluded that the nanocomposite PEG-4MAL microgel is a promising intra-articular vehicle for tissue-localised drug delivery in the knee bone. Similarly, Holyoak et al. (2019) have characterized a PEG-4MAL hydrogel system as a “mechanical pillow” to cushion weight-bearing joints, withstand repetitive loads and enhance intra-articular injection of dexamethasone-containing nanoparticles for the treatment of a mouse model of osteoarthritis of the knee. It was shown that the PEG-4MAL hydrogel maintained its mechanical properties after physiologically relevant cyclic compression and was more resistant to loading *in vitro*. In an *in vivo* load-induced KOA mouse model, the PEG-4MAL hydrogel acted as a mechanical pillow that protected the knee joint from cartilage degradation and inhibited the formation of osteoid.

**FIGURE 7:**
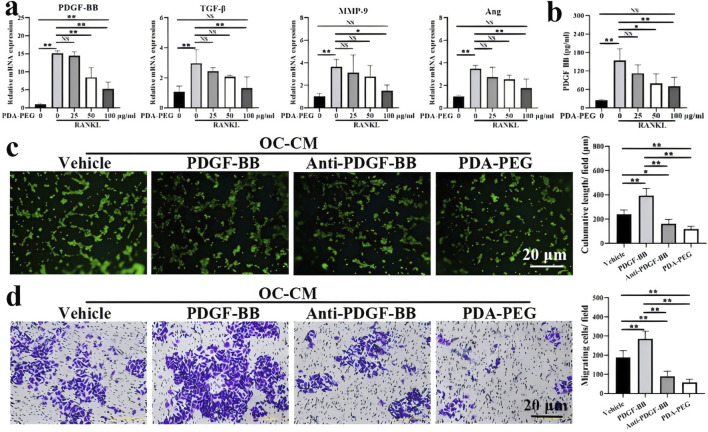
**(a)** Expression of angiogenesis-related genes PDGF-BB, TGF-β, MMP-9 and Ang detected by qRT-PCR; **(b)** Quantification of PDGF-BB determined by ELISA; **(c)** Effect of PDA-PEG NPs on T-tubule formation; **(d)** Effect of PDA-PEG NPs on cell migration (n = 3) Notes: ‘*’ indicates p < 0.05, “**” indicates p < 0.01, and NS indicates not significant. CC BY 4.0. Copyright 2022 The Author(s).

In contrast to the above, Wu et al. (2022) synthesised methoxypolyethylene glycol amine-modified polydopamine-polyethylene glycol nanoparticles (PDA-PEG NPs) for the treatment of early-stage KOA. The PDA-PEG NPs could inhibit osteoclastogenesis by modulating the nuclear factor-kappa B and mitogen-activated protein kinase signalling pathways. The results showed that stimulation of receptor activators of nuclear factor-kappaB ligands promoted the expression of platelet-derived growth factor-BB (PDGF-BB), TGF-β, MMP-9, and Ang, especially at high concentrations, which was inhibited by PDA-PEG NPs (Figure 7a). In addition, the concentration of PDGF-BB in osteoblast conditioned medium (OC-CM) was 153.44 ± 38.88 pg/ml, whereas PDA-PEG NPs showed a significant dose-dependence on the concentration of PDGF-BB (Figure 7b). The tube length of human umbilical vein endothelial cells after OC-CM treatment was 238.10 ± 36.83 μm, and the test tube length was enhanced after exogenous PDGF-BB treatment (Figure 7c). The results of migration experiments indicated that PDA-PEG NPs could inhibit OC-CM-induced angiogenesis by regulating the key proangiogenic factor PDGF-BB (Figure 7d).

In addition, Zeng et al. (2021) used functionalized nanographene oxide (NGO) with linear chain amine capped PEG and branched polyethyleneimine (BPEI) to synthesize biocompatible NGO-PEG-BPEI (PPG) scaffolds. Using PPG with KGN for intracellular delivery with BMSCs, KGN-enhanced BMSCs were obtained by simple mixing and co-incubation with BMSCs for the treatment of osteoarthritis symptoms in the knee in rats. *In vitro* studies demonstrated rapid uptake of PPG within the first 4 h after incubation, reaching saturation at 12 h. *In vivo* studies demonstrated that PPG-KGN-treated BMSCs helped to prevent knee joint space narrowing, pathological mineralisation, KOA development and pain, and improved periprosthetic soft tissue regeneration. Li and Yang (2024) used both PCL with PEG azide as a block polymer and β-cyclodextrin and adamantane as a host-guest assembly, and the object was constructed as a self-repairing chitosan hydrogel and a dispensed compound drug encapsulation system for the repair and treatment of knee osteoarthritis in rats. It was shown that the developed hydrogel could simultaneously inhibit osteoclastogenesis and induce chondrogenesis, and this dual crosslinked encapsulation system could lubricate the surface of the knee joint and prevent wear and tear during exercise. In contrast to the above, Zerrillo et al. (2021) developed novel trifluoroacetamide (TFA)-modified polymer NPs as carriers for multimodal nanoprobes and loaded them with a NIR dye, and characterised the generated PLGA-PEG-TFA NPs *in vitro* and *in vivo* in a KOA mouse model using the C28/I2 human chondrocyte cell line. Dye-loaded NPs were injected into mouse knee joints and then observed and tracked *in vivo* by fluorine-19 MRI and fluorescence imaging. The results of the study showed that the NPs were well absorbed and non-toxic to cells as confirmed by confocal microscopy. It suggests that this intra-articular drug delivery system has a good therapeutic effect in repairing osteoarthritis of the knee in mice.

In summary, the impact of PEG in the treatment of osteoarthritis of the knee is mainly reflected in its role as an excipient of drug preparation. By enhancing the solubility, stability and bioavailability of drugs, PEG can indirectly promote the therapeutic effect. PEG has good solubility, which can help certain insoluble drugs to dissolve better in the preparation process, thus ensuring the effective release and absorption of drugs in the body. However, the specific therapeutic effect needs to be evaluated in combination with other components of the drug and the patient’s specific situation.

### 2.5 Effect of other synthetic polymer materials on the treatment of knee osteoarthritis

In addition to the different biodegradable synthetic polymer materials summarised above for the repair of osteoarthritis of the knee bone, there are other synthetic polymer materials used for the treatment of osteoarthritis of the knee bone that have been reported (Table 1), such as polytrimethylene carbonate (PTMC), polyvinyl alcohol, nanocrystal-polymer particles (NPPs), poly(ethylene oxide) (PEO) and poly(propylene oxide) (PPO), Poly (3-hydroxybutyrate-co-ε-caprolactone) copolymers (PHBCL), poly(propylene sulfide) (PPS) and other materials. At this stage, some synthetic polymer materials can be used well in combination with drugs to form a polymer-borne drug delivery system. The drug delivery factors are continuously released in the focal area around the knee joint to achieve the effect of inhibiting the inflammatory factors.

**TABLE 1:** Synthetic polymer materials for the treatment of knee osteoarthritis.

Materials	Drug	In vivo/vitro	Experimental Subject	Main findings	References
Acid-activable PAE	Monoidoacetic acid	In vivo, In vitro	Rat	Acid-activatable curcumin polymer micelles well inhibit TNF-α and IL-1β	Kang et al. (2020)
mPEG-PTMC	Oligopeptide	In vitro	-	Nanoparticles have a temperature sensitivity between 30°C and 40°C	Wang et al. (2020)
NPs	Epidermal growth factor receptor	In vivo, In vitro	Rat	TGFɑ-NPs attenuate postoperatively induced KOA cartilage degeneration and joint pain	Wei et al. (2021)
PLGA, PVA	CCR2	In vivo, In vitro	Rat	Sustained release of CCR2 inhibitors *in vivo* for more than 21 days	Ozkan et al. (2023)
PLA, PVA	Etoricoxib	*In vitro*	-	3% w/w PVA formulation has the smallest particle size and longest drug release	Fan et al. (2023)
NPPs	p38α/β MAPK inhibitor	In vivo, In vitro	Rat	The drug loading was about 31.5% (w/w) and about 20% of the PH was released in 3 months	Maudens et al. (2018b)
Epichlorohydrin crosslinked prepolymer	Hydrocortisone, Ttriamcinolone,Dexamethasone	*In vitro*	-	Through this “affinity-based drug delivery,” the study demonstrated that the delivery rate of solid polymers can be extended from hours and days to weeks and months	Rivera-Delgado et al. (2018)
PEO-PPO-PEO	Recombinant adeno-associated virus	*In vivo*, *In vitro*	Human	Based on the controlled therapeutic properties of PEO-PPO-PEO micelles, resulting in increased proteoglycan deposition and stimulation of cell proliferation index in chondrocytes of KOA patients	Rey-Rico et al. (2018)
PHBCL	Diclofenac sodium	*In vitro*	-	MTT tests showed no toxic effects of diclofenac sodium at the required concentrations	Musumeci et al. (2020)
PPS	Micro-spheres (MS)	*In vivo*, *In vitro*	Rat	PPS-MS scavenged hydrogen peroxide and hypochlorite *in vitro* and reduced matrix metalloproteinase activity *in vivo*	Kavanaugh (2017)

In summary, polymers with higher molecular weights degrade more slowly because the larger molecules take more time to hydrolyse and break into smaller molecules. Therefore, the rate of degradation can be controlled to some extent by selecting the appropriate molecular weight of the polymer. In addition, the rate of hydrolysis and biodegradability can be regulated by changing the functional groups in the polymer chains, the degree of cross-linking and the rigidity of the chains. Some scholars before us will use catalysts or inhibitors that can accelerate or slow down the degradation process of polymers. By precisely controlling the type and amount of these additives, the degradation properties of the material can be further optimized.

## 3 Natural polymer-based drug delivery systems

Some natural biomedical materials used in the treatment of osteoarthritis of the knee do not produce toxicity or side effects, and have good compatibility and therapeutic efficacy in the treatment of osteoarthritis of the knee. Natural polymeric materials, such as collagen, filipin, and hyaluronic acid, are widely used as scaffolding materials for tissue engineering. These natural polymeric materials can be easily chemically modified to introduce targeting factors or stimuli-responsive moieties to achieve controlled release of drugs under specific conditions. Regarding the drug delivery mechanism, natural polymers can be bound to drugs through physical encapsulation, chemical binding (polymer-drug concatenates) or self-assembled structures. In addition, these materials can provide the necessary space and support for chondrocyte attachment, proliferation and secretion of substrates by processing them into 3D porous scaffolds, hydrogel forms. Natural polymer materials piggybacking drug delivery systems show great potential in repairing osteoarthritis of the knee.

### 3.1 Effect of collagen on the treatment of knee osteoarthritis

Collagen is a commonly used natural polymer material that is widely used in cartilage repair. Studies have shown that these materials have good biocompatibility and mechanical properties, support cartilage cell growth and differentiation, and promote the synthesis and secretion of cartilage matrix. By processing these materials into forms such as three-dimensional porous scaffolds or hydrogels, they can provide an ideal microenvironment for cartilage repair.

A study was conducted to evaluate the symptoms and biomarkers of cartilage degradation in the knee joints of patients with osteoarthritis of the knee by evaluating oral natural type II collagen therapy in patients with osteoarthritis of the knee when administered concomitantly with acetaminophen. The study showed significant improvements in joint pain, Western Ontario and McMaster Universities Osteoarthritis Index (WOMAC) and quality of life in the combination group compared to baseline, with significant differences in visual analogue scale (VAS) walking scores. *In vivo* results found no significant improvement in urinary biochemical markers of cartilage degradation in any group. In conclusion, natural type II collagen therapy combined with acetaminophen has shown significant efficacy in the treatment of patients with osteoarthritis of the knee (Bakilan et al., 2016). It has been shown that the WOMAC and VAS score outcome measures show better outcomes with undenatured type II collagen (UC-II). Walking measurements improved significantly from baseline, as reflected by improved timed jumping and 6-minute walk tests (Kumar et al., 2023). As above, Lugo et al. (2015) evaluated the efficacy and tolerability of UC-II for the treatment of pain and related symptoms in patients with KOA by comparing it with placebo and glucosamine hydrochloride plus chondroitin sulphate (CS). The results of the study showed that at the 180th day, the UC-II group showed a significant reduction in the total WOMAC score compared to placebo (P = 0.002) and GC (P = 0.04). Sadigursky et al. (2022) similarly used UC-II for the treatment of pain and consequently improvement of knee function in 60–80 years old females with osteoarthritis of the knee. The results of the study showed a significant improvement in the quality of life in the physical domain in the treated group compared to the control group. In addition, there was a difference between the first and last assessments in the pain visual analogue scale and WOMAC scores. Cahue et al. (2007) determined collagen synthesis by a commercial enzyme-linked immunosorbent assay (ELISA) for the detection of type II collagen c-peptide and also serum markers of collagenase cleavage of cartilage type II collagen. Knee radiographs were taken at baseline and at 18 months. It was found that baseline levels of these markers, considered alone, were not associated with changes in the odds of progression. Garnero et al. (2002) measured serum levels of type IIA procollagen N-precursor peptide (PIIANP) and urinary excretion of type II collagen C-terminal crosslinked telopeptide (CTX-II) as markers of type II collagen synthesis and degradation, respectively. Studies have shown that patients with low levels of PIIANP and high levels of CTX-II have joint damage that progresses 8 times faster than other patients. The combination of these two new markers, serum PIIANP and urinary CTX-II, could be used to identify KOA patients at high risk for rapid progression of joint damage. In addition, Altindag et al. (2007) assessed serum antioxidant status by measuring total antioxidant capacity (TAC), thiol levels, catalase activity, and total peroxides (TP) in patients with osteoarthritis and healthy controls to calculate oxidative stress index (OSI). The study showed that prolylase activity was negatively correlated with TP and OSI and positively correlated with TAC. The results of the study showed that patients with osteoarthritis have increased oxidant parameters and decreased antioxidant parameters which inhibit the progression of osteoarthritic disease. Furuzawa‐Carballeda et al. (2009) co-cultured cartilage and synovial tissues from KOA patients with or without 1% polymerised collagen for 7 days and determined the supernatant by ELISA for of pro- and anti-inflammatory cytokines in the supernatant by ELISA. The results showed that polymerised type I collagen induced a 3-fold–6-fold increase in cell proliferation, proteoglycan content and type II collagen expression, and inhibited IL-1β and TNF-α production. However, an investigation into the efficacy and safety of natural type II (TII) collagen in patients with knee OA found that the TII collagen group significantly improved overall joint health compared to the placebo group, with statistically significant effects observed as early as 4 weeks after administration of the study product. Not only did TII Collagen provide significant rehabilitative therapeutic benefits and a greater safety profile for KOA, but it also improved the quality of life of patients (Luo et al., 2022).

In addition, De Luca et al. (2019) used *in vitro* and *in vivo* results by using a novel injectable collagen preparation (ChondroGrid) consisting of bovine hydrolysed <3 kDa type I collagen for the treatment of symptoms of osteoarthritis of the knee in humans. The results of the study found that Bern scores were higher when cells were cultured with ChondroGrid. Patients experienced a 44% Lequesne score and a 55% VAS at reduced mobility scores.The study suggests that ChondroGrid may induce chondrocytes to produce hyaline cartilage, prevent fibrous tissue formation and be a safe and effective adjunct to the treatment of symptomatic knee osteoarthritis. He et al. (2019) demonstrated the effectiveness of ChondroGrid in the treatment of symptomatic knee osteoarthritis by developing a specific immunoassay for the newly discovered collagen type X (CoL10)-type neoepitopes for blood quantification to assess their diagnostic value for radiological KOA. The immunohistochemical detection of this new epitope was performed on human KOA cartilage. The type X collagen neo-epitope (CoL10neo) was developed and quantified in two clinical studies. The results show that the new epitope generated by histone K is localised in the pericellular matrix of the chondrocytes, whereas it is more degraded and higher levels of CoL10neo values can be observed in the subjects. The study demonstrates that CoL10neo associated with hypertrophic chondrocytes can be used as a diagnostic biochemical marker for KOA.

### 3.2 Effect of chondroitin sulphate on the treatment of knee osteoarthritis

CS is one of the main components of articular cartilage and has the ability to lubricate joints, reduce friction, and promote cartilage repair. Studies have shown that by injecting chondroitin sulphate natural polymer material, it can improve the clinical symptoms of patients with osteoarthritis of the knee, reduce pain, swelling and stiffness, and improve the quality of life of patients.

Bruyère and Reginster (2024) demonstrated superiority over placebo in reducing pain and improving function in patients with symptomatic knee osteoarthritis over a 6-month period via 800 mg/day drug grade CS. Lasisi et al. (2024) divided the research trial into three groups, with participants in the first group receiving 1 g of glucosamine sulphate via iontophoresis, and those in the second group receiving 1 g of chondroitin sulphate iontophoresis using a transurethral electrode technique, placed twice a week for 12 weeks. Participants in the third group received the intervention in the form of quadriceps strengthening exercises. The results of the study showed that all three intervention modalities significantly reduced pain in all groups after 12 weeks (P = 0.001) and improved patients’ functional activity levels and range of motion. In addition, it has been suggested that the nonsteroidal anti-inflammatory drug (NSAID) celecoxib is often prescribed to treat patients with KOA, but carries significant gastrointestinal and cardiovascular risks. Chondroitin sulphate in combination with glucosamine has been favoured by patients as an alternative to NSAIDs and has been clinically effective (Eriakha et al., 2024). Dardenne et al. (2024) mapped the Lequesne index to the EuroQol 5-dimensional (EQ-5D-5L) utility index for patients with knee osteoarthritis. The results of the study showed that two of the models combining age, gender, with or without body mass index, and the Lequesne index demonstrated the best fit in the regression. Effectively translating the Lequesne index into EQ-5D-5L values for patients with osteoarthritis of the knee provides a valuable tool for clinicians in the treatment of osteoarthritis of the knee disease. Minoretti et al. (2024) divided HA, glucosamine (GLc), and CS into three groups according to the equal distribution, and all the participants received pain relief, improvement in functional capacity, and improvement in serum adropin levels, both at baseline and after a 4-week intervention period. and serum adropin levels (An emerging biomarker of KOA). The results of the study showed that the HA group was superior to the GLc + CS group in relieving resting pain, motor pain, and the WOMAC pain subscale, and serum adropin levels were significantly higher in both groups compared to baseline. Oral supplementation with HA or GLc + CS is potentially beneficial for symptom control in patients with mild to moderate KOA. Muftic et al. (2024) administered Cartinorm (1,500 mg glucosamine sulphate, 800 mg chondroitin sulphate) orally to 60 patients with clinical and imaging signs of osteoarthritis of the knee and tested each patient on a pain scale and Ostwestry index. The results of the study showed a statistically significant reduction in pain as assessed by the VAS Pain Scale on the first day and at the end of the three consecutive months of the study, indicating an improvement in the quality of life after 3 months of Cartinorm administration. The study demonstrates that the use of Cartinorm has a significant therapeutic effect on patients with KOA, helping to maximise the patient’s return to family and society.

However, Clegg et al. (2006) investigated this by randomly assigning 1,583 patients with symptomatic osteoarthritis of the knee to receive 1,500 mg of Glucosamine(GA) and 1,200 mg of chondroitin sulphate, GA and chondroitin sulphate, 200 mg of celecoxib per day, or placebo for 24 weeks. The study found that response rates were 3.9 percentage points higher for GA, 5.3 percentage points higher for chondroitin sulfate, and 6.5 percentage points higher for the combination therapy compared to response rates in the placebo group. GA and chondroitin sulfate were not significantly better than placebo in reducing knee pain. As same as above, Babur et al. (2022) used short term supplementation of GA and chondroitin sulphate in combination with manipulative therapy and resistance exercise training in 24 patients with KOA, subjects were randomly assigned to active control group A and experimental group B. It was found that no significant difference in any of the outcome indicators was observed between the two groups at 2 weeks and 4 weeks post intervention. Manipulative therapy and resistance exercise training were effective in the treatment of knee osteoarthritis, however, supplementation with glucosamine and chondroitin sulphate for 4 weeks did not show additional benefit. Fransen et al. (2015) observed joint space narrowing (JSN) and pain in patients with chronic KOA pain by administering glucosamine sulfate 1,500 mg and chondroitin sulfate 800 mg orally. The results of the study showed that allocation to the dietary supplement combination (Glucosamine-Chondroitin) resulted in a statistically significant reduction in JSN at 2 years compared to placebo. Some scholars had patients undergo oral administration of GA 500 mg and CS 400 mg capsules of the drug, starting with 3 capsules per day for 3 weeks according to the approved patient information instructions, and observed the patients’ Knee Injury and Osteoarthritis Outcome Score (KOOS). The results of the study showed that all subscale scores of the KOOS (Pain, Symptoms, Function and Quality of Life) showed clinically and statistically significant improvements. The study concluded that long-term oral GA + CS can reduce pain levels in patients with KOA in routine clinical practice (Lila et al., 2023). Furthermore, Sevimli et al. (2021) have applied a cruciate ligament cutting model to New Zealand rabbits to induce experimental osteoarthritis. The control group was fed standard rabbit diet and the other groups were additionally fed 17 mg/kg/day CS/microbial chondroitin sulphate (MCS) for 12 weeks. The results of the study showed that statistically significant regenerated tissue was observed in the histopathological cartilage tissue as compared to the control group of CS given to osteoarthritic rabbits from different sources and the group with the highest chondroprotective effect was the MCS derived from *Escherichia Coli*.

### 3.3 Effect of other natural polymer materials on the treatment of knee osteoarthritis

Hydrogel (HG) has a three-dimensional network structure, and this structure not only provides the necessary space for cells to survive, but also mimics the natural ECM, which provides cells with conditions similar to the natural microenvironment. In the treatment of knee osteoarthritis, hydrogel can fill in the joint cavity and provide physical support, while its lubricating properties can reduce friction on the joint surfaces and reduce cartilage wear, thus relieving pain and improving joint function. HA has free radical scavenging properties, which help reduce inflammatory responses within the joints. In osteoarthritis of the knee, inflammation is one of the major causes of joint pain and swelling. By injecting HA, the inflammatory response within the joints can be reduced and the release of inflammatory mediators can be reduced, thus relieving the symptoms of pain and swelling. HA injections for knee osteoarthritis are widely applicable, especially for patients with mild and moderate knee osteoarthritis. However, in patients with severe or advanced osteoarthritis of the knee, the therapeutic effect of HA may be suboptimal due to severe disruption of the intra-articular environment and significant cartilage damage. Sodium alginate (SA) works in the treatment of osteoarthritis of the knee mainly by combining with specific drugs or materials. Silk protein (SF) hydrogels have excellent biocompatibility and can support the growth of chondrocytes and provide a favourable environment for chondrocytes to survive. Silk protein hydrogels have excellent mechanical properties and can withstand certain mechanical loads while maintaining their structural stability. Stem cells have the ability to fight inflammation and regulate immune response, and are able to secrete a variety of growth factors and anti-inflammatory factors to reduce inflammatory mediators in the joints. Stem cell therapy can help to slow down the progression of osteoarthritis of the knee. Other natural biomedical materials commonly used at this stage for the treatment of knee osteoarthritis, such as hydrogel, hyaluronic acid, sodium alginate, and filipin protein are summarised in (Table 2).

**TABLE 2:** Natural polymer materials in different carriers for the treatment of KOA.

Materials	Drug/Cell	*In vivo*/vitro	Experimental Subject	Main findings	References
HA,HG	Heparin	*In vivo*, *In vitro*	Human	Significant improvement in the WOMAC index pain subscale 6 months after injection	Russu et al. (2021)
HA,HG	Hymovis MO.RE	*In vivo*, *In vitro*	Human	Injection of Hymovis MO.RE. (32 mg/4 mL) dose has excellent effect on osteoarthritis of knee joints	Bernetti et al. (2021)
SA,HA	Icariin	*In vivo*, *In vitro*	Rat	Extracellular vesicles and ICA promote proliferation and migration aspects of knee chondrocytes	Li et al. (2024b)
SA	human MSCs (hMSCs)	*In vivo*, *In vitro*	Rat	SA-enveloped hMSCs reduced articular cartilage degeneration and subchondral bone remodelling	McKinney et al. (2022)
CS	decellularized Extracellular matrix-chitosan (dECM)	*In vivo*, *In vitro*	Rat	dECM upregulates cartilage synthesis and metabolism-related genes	Chen et al. (2021)
HA	Gold nanoparticles	*In vivo*, *In vitro*	Rat	Lower OARSI and synovitis scores and more collagen type II deposits	Chen et al. (2025)
SF	platelet rich plasma (PRP)	*In vivo*, *In vitro*	Human	SF-PRP hydrogel can be easily injected, biodegraded and continuously releases growth factors	Jiraboonsri et al. (2025)
SF	Curcumin	*In vivo*, *In vitro*	Rat	Gelatin/SF (30/70) microspheres delayed cell destruction in joint and synovial tissues after 8 weeks and showed a long-lasting anti-inflammatory effect	Ratanavaraporn et al. (2017)
SF	MSCs	*In vivo*, *In vitro*	Rat	Masson staining and Mankin score assessment confirmed the formation of new cartilage within 8 weeks	Chen et al. (2023)
SF,HA	hMSCs	*In vitro*	-	SF/HA scaffolds display a spherical structure of cells in a porous structure that promotes cartilage production	Jaipaew et al. (2016)

In conclusion, hydrogel mainly relieves pain and improves joint function through its scaffolding, lubrication and drug delivery functions; sodium alginate promotes cartilage regeneration and repair by combining with specific drugs or materials; and filipin protein is an ideal material for constructing cartilage-like organs due to its excellent biocompatibility and mechanical properties, and offers new possibilities for personalised treatment. Stem cells can differentiate into chondrocytes to fill cartilage defects, increase the smoothness of joint surfaces, and reduce friction and wear. However, it should be noted that the application of these materials in the treatment of osteoarthritis of the knee is still in the research stage and has not yet been widely used in clinical practice. Natural polymer materials can be chemically or physically modified to improve their degradation rate and biocompatibility. Some biodegradable polymers are broken down by microorganisms into carbon dioxide, water and other inorganic products, which are generally safe for living organisms. However, not all degradation products of polymers are non-toxic. The decrease in PH and change in osmotic pressure in the surrounding environment during polymer degradation may impair the function of macrophages and fibroblasts and weaken tissue defences. Therefore, the optimal application method, dosage, and long-term safety of these materials need to be further explored in future studies to promote their widespread use in the treatment of knee osteoarthritis.

## 4 Conclusion and outlook

Effective treatment of osteoarthritis of the knee has been a major clinical problem, as traditional medications have made it difficult to achieve excellent therapeutic and restorative outcomes. The use of safe and biodegradable polymer materials as carriers to deliver therapeutic drugs or stem cells to the appropriate focal areas can be an effective treatment for osteoarthritis of the knee. In summary, we have learned that in the treatment of KOA, drugs with polymer properties may be used, and this polymer property enables the slow release of drugs to form a protective film in the joint cavity, reducing the friction and wear of the joint surfaces, and thus relieving the pain and swelling of KOA patients. However, not all KOA therapeutic drugs have drug loading capacity. Some oral medications typically work by entering the body via the oral route without the need for an additional drug carrier. In contrast, chondro-nutrients promote cartilage repair and regeneration by providing the nutrients needed by chondrocytes. The size of a KOA therapeutic drug usually refers to the molecular weight, particle size, or formulation form of the drug, which affect the solubility, stability, bioavailability, and pharmacokinetic properties of the drug. In addition, the shape and size of the injection needle used in intra-articular injection therapy can also affect the effectiveness of the drug injection and patient comfort. In this review, we collected and analysed studies applying different synthetic polymer materials and natural polymeric materials for the treatment of KOA in clinical trials (Table 3).

**TABLE 3:** Drug delivery systems with clinical approval for different polymer carriers for the treatment of KOA.

Formulation type	Materials	Active agent	*In vivo*/vitro	Model type	Treatment mechanism	References
Synthetic polymers	PCL	Lignin	In vivo, In vitro	Rabbit	The nanofibre membrane is biocompatible and biodegradable and provides sustained antioxidant activity	Gu et al. (2022), Liu et al. (2019a)
	PCL-PEG-PCL	Flurbiprofen	*In vivo*, *In vitro*	Rat	Flurbiprofen is sustained *in vivo* from thermal gels for more than 3 weeks	Salama et al. (2020)
	PLA	Triamcinolone acetonide	*In vivo*, *In vitro*	Rat	Advantages of using sustainable polymers for effective drug delivery in new combinations with favourable therapeutic effects for the treatment of KOA in rats	Fan et al. (2022), Ho et al. (2019)
	PLGA	Betamethasone sodium phosphate	*In vivo*, *In vitro*	Rat	A single injection of 30 mg of PLGA-nanosteroids resulted in almost complete resolution of the inflammatory response after 1 week	Higaki et al. (2005), Horisawa et al. (2002)
	PLGA	Melatonin	*In vivo*, *In vitro*	Rat	MT@PLGA-COLPB can inhibit the TLR2/4-MyD88-NFκB signalling pathway through inhibition	Liang et al. (2023)
	PLGA-PEG-PLGA	Etoricoxib	*In vivo*, *In vitro*	Rat	NPs loaded with etoricoxib showed sustained drug release *in vitro* for more than 28 days	Liu et al. (2019b)
	PEG-4MAL	Rhodamine B	*In vivo*, *In vitro*	Rat	PEG-4MAL microgels were retained in the knee joint space of rats for at least 3 weeks without inducing any degenerative joint changes	Mancipe Castro et al. (2020)
Natural polymers	Type II Collagen	Paracetamol	*In vivo*	Human	*In vivo* results revealed no significant improvement in biochemical markers of cartilage degradation in urine in any group	Bakilan et al. (2016)
	Undenatured type II collagen	Chondroitin sulfate	*In vivo*	Human	WOMAC and VAS score outcome indicators showed better outcomes in the UC-II group	Kumar et al. (2023), Lugo et al. (2015), Sadigursky et al. (2022)
	CS	Celecoxib	*In vivo*	Human	Celecoxib can be used as an alternative to NSAIDs and has shown promising therapeutic results in clinical practice	Kumar et al. (2023), Lugo et al. (2015), Sadigursky et al. (2022), Kumar et al. (2023), Lugo et al. (2015), Sadigursky et al. (2022)
	CS	Cartinorm	*In vivo*	Human	Assessment of pain on the VAS Pain Scale at the beginning and end of the patient’s 3 consecutive months showed a statistically significant reduction in pain	Kumar et al. (2023), Lugo et al. (2015), Sadigursky et al. (2022)

Currently, different types of biodegradable polymers are used to form a drug delivery system to the body, through the continuous release of the drug and the degradation rate of the polymer material in the body to achieve the best results in the treatment of osteoarthritis of the knee. Biodegradable systems have the advantages of environmental protection, resource conservation, safety and health, but they also have the disadvantages of performance defects, processing difficulty, difficulty in controlling the degradation time, and higher prices. Biodegradable systems have limitations in terms of degradation conditions, production costs, performance and service life. In clinical applications, the choice of whether to use biodegradable systems needs to be made according to specific needs and conditions. Natural and synthetic biodegradable material systems are capable of being broken down by microorganisms in the natural environment and eventually converted into harmless small molecules. The research, development and application of natural and synthetic biodegradable materials promote the development of green industries and the transformation and upgrading of the economy. In KOA treatment, if the drug is administered in the form of polymer microspheres, gels or films, the solubilisation effect will directly affect the release kinetics of the drug, and hence the therapeutic effect. The degradation rate of the polymer needs to match the release rate of the drug to ensure a sustained and stable release of the drug into the joint cavity for optimal therapeutic efficacy. On the contrary, the swelling and degradation process of polymers may produce some harmful substances that may cause irritation or damage to the joint tissues. Therefore, when selecting a polymer carrier, its biocompatibility needs to be evaluated to ensure that the drug works safely and effectively in the body. However, there are still questions that need to be further researched and discussed, such as whether the release of the drug will produce side effects? How to control the dosage size of the drug effectively? Does the polymer material have toxicity and produce acidic substances in the body? The above questions are still to be further solved and researched. The ultimate goal of the experimental research is to benefit mankind and to be safely and effectively applied in clinical rehabilitation.

As materials science continues to develop, more novel biodegradable materials will be developed. These materials may have properties such as better biocompatibility, stronger drug loading ability, and more controllable degradation rate, providing more options for KOA therapy. Combined with intelligent technologies, intelligent drug delivery systems can be developed. Biodegradable polymer materials offer new ideas for implant design. By changing the composition and structure of materials, implants with specific shapes, sizes and functions can be prepared to meet different clinical needs. With the development of advanced manufacturing technologies such as 3D printing, biodegradable polymer materials can be personalised. Clinicians can design the most suitable shape and size of the implant according to the patient’s specific situation and needs, thus improving treatment results and patient satisfaction. These systems can automatically adjust the rate of drug release and dosage according to the patient’s physiological indicators to achieve more precise treatment. With the continuous progress of material science, medical technology and intelligent technology, more innovative therapeutic methods and products will be developed to bring better treatment results and quality of life to KOA patients. Therefore, the continuous innovation of synthetic and natural polymeric materials for drug delivery systems has brought great hope and benefits for the treatment of patients suffering from osteoarthritis of the knee. We believe that through continuous scientific research, these innovative polymer-based drug or cell-based therapeutic prescriptions will soon be available for clinical treatment and rehabilitation applications.
